# Systematic review of prognostic models for recurrent venous thromboembolism (VTE) post-treatment of first unprovoked VTE

**DOI:** 10.1136/bmjopen-2016-011190

**Published:** 2016-05-06

**Authors:** Joie Ensor, Richard D Riley, David Moore, Kym I E Snell, Susan Bayliss, David Fitzmaurice

**Affiliations:** 1 Research Institute of Primary Care and Health Sciences, Keele University, Staffordshire, UK; 2 Public Health, Epidemiology and Biostatistics, Institute of Applied Health Research, University of Birmingham, Birmingham, UK; 3 Primary Care Clinical Sciences, Institute of Applied Health Research, University of Birmingham, Birmingham, UK

**Keywords:** Venous thrombosis, Recurrence risk, Prognostic model, Prediction model

## Abstract

**Objectives:**

To review studies developing or validating a prognostic model for individual venous thromboembolism (VTE) recurrence risk following cessation of therapy for a first unprovoked VTE. Prediction of recurrence risk is crucial to informing patient prognosis and treatment decisions. The review aims to determine whether reliable prognostic models exist and, if not, what further research is needed within the field.

**Design:**

Bibliographic databases (including MEDLINE, EMBASE and the Cochrane Library) were searched using index terms relating to the clinical field and prognosis. Screening of titles, abstracts and subsequently full texts was conducted by 2 reviewers independently using predefined criteria. Quality assessment and critical appraisal of included full texts was based on an early version of the PROBAST (Prediction study Risk Of Bias Assessment Tool) for risk of bias and applicability in prognostic model studies.

**Setting:**

Studies in any setting were included.

**Primary and secondary outcome measures:**

The primary outcome for the review was the predictive accuracy of identified prognostic models in relation to VTE recurrence risk.

**Results:**

3 unique prognostic models were identified including the HERDOO2 score, Vienna prediction model and DASH score. Quality assessment highlighted the Vienna, and DASH models were developed with generally strong methodology, but the HERDOO2 model had many methodological concerns. Further, all models were considered at least at moderate risk of bias, primarily due to the need for further external validation before use in practice.

**Conclusions:**

Although the Vienna model shows the most promise (based on strong development methodology, applicability and having some external validation), none of the models can be considered ready for use until further, external and robust validation is performed in new data. Any new models should consider the inclusion of predictors found to be consistently important in existing models (sex, site of index event, D-dimer), and take heed of several methodological issues identified through this review.

**PROSPERO registration number:**

CRD42013003494.

**Strengths and limitations of this study**

- To our knowledge, this is the first systematic review identifying prognostic models for venous thromboembolism recurrence risk in the unprovoked population, using a robust systematic methodology.
- The study is also the first to assess the validity of the existing models in terms of risk of bias and applicability.
- We were unable to perform a quantitative analysis of the identified articles due to a lack of homogeneity in many areas, including the predictors used, model types and study populations.
- All models require further independent external validation, and as such the true performance of the models in the wider unprovoked population must be assessed in new research.

## Introduction

Venous thromboembolism (VTE) is the third most common cardiovascular disease after heart attack and stroke; it is a chronic condition with estimated incidence at 1 per 1000 person years.^1–3^ VTE often presents as deep vein thrombosis (DVT), with some patients suffering an embolism in the lungs known as a pulmonary embolism. An initial VTE developed in the presence of a known provoking factor may be termed ‘provoked’, while those developed in the absence of clinical risk factors may be termed ‘unprovoked’.^3^ ^4^ There are several known predisposing risk factors including surgery, trauma, hormone intake, pregnancy and prolonged immobility.^3^ ^5^ Such factors can be considered as acquired risk factors, because they are transient, that is, while they increase the risk of an initial VTE, they are temporary, and when the provoking factor is removed, the patient is at a low risk of recurrence, for example, postsurgery.^3–5^

The aim of therapy for VTE is twofold, initially to prevent extension of the acute thrombosis, and secondarily to prevent both recurrence and long-term sequelae such as post-thrombotic syndrome and pulmonary hypertension. Current treatment comprises initial management with heparin, usually low molecular weight heparin for a minimum of 5 days, overlapping with oral anticoagulant (OAC) therapy (usually warfarin in the UK) until the international normalised ratio is above two. It is usual to treat an initial VTE for a minimum of 3 months; however, the optimum duration of therapy beyond this is unclear.^6^ ^7^ Treatment with novel oral anticoagulants is a new alternative treatment to heparin and warfarin.

Owing to the transient nature of provoking factors, patients with a first unprovoked VTE are at much higher risk of recurrent VTE (approaching 30% at 5 years after cessation of therapy) as the cause is unknown.^3^ ^5^ Prevention of recurrent VTE poses a difficult clinical decision problem; a balance must be struck between the risks of recurrent thrombosis if anticoagulant treatment is stopped versus the risks of bleeding associated with continued anticoagulation therapy.^3^ ^6^

Therefore, it is important to identify individuals with a high risk of VTE recurrence compared with the risk of major bleeding on anticoagulation, in order to inform treatment strategies. However, the population of patients with unprovoked VTE is highly heterogeneous and risk of VTE recurrence varies considerably across individuals.^8–10^ Therefore, there is much interest in developing prognostic models for VTE recurrence. A prognostic model is a statistical equation that predicts an individual's outcome risk based on the combination of their values of multiple predictors (eg, age, sex, biomarkers).^11^ A key stage of prediction model research is model development. This uses a data set to identify important predictors and then develops the model equation; it usually also examines the model's apparent performance in this same data, possibly using resampling techniques to adjust for optimism (internal validation). The next stage is external validation, which uses data external to the model development data and its source, and examines whether the model predictions are accurate in another (related) setting. External validation is crucial as model performance is usually overoptimistic when considered only in the development data set.^11–13^ Validation typically focuses on discrimination performance (ie, the model's ability to separate those with and without the outcome) and calibration performance (ie, the agreement between the model's predicted risk and the observed outcome risk).

### Aims of the review

A reliable prognostic model is needed for the unprovoked VTE population, in order to inform clinical and patient decision-making with regard to treatment strategies,^14^ in particular whether or not to extend treatment beyond the initial period (eg, 3 months) with OACs to prevent recurrent VTE.

The aim of this systematic review was to identify and summarise studies developing or validating a prognostic model for individual VTE recurrence risk following cessation of therapy for a first unprovoked VTE. Through the identification of existing studies, the review will help to determine whether reliable prognostic models exist and, if not, what further research is needed within the field. In particular, the review appraises the evidence for and against each existing model, to help clinicians and other practitioners to better understand their strengths and weaknesses,^14^ allowing more informed decisions to be made on which (if any) models to use in practice. A protocol for the review was registered with PROSPERO (CRD42013003494) and published in Systematic Reviews.^15^

## Methods

### Search strategy

The following bibliographic databases were searched: Cochrane Library (Wiley; including the Cochrane Database of Systematic Reviews, DARE, HTA Databases and CENTRAL Register of Controlled Trials), MEDLINE (Ovid) 1950 to July 2014, MEDLINE In—Process & Other Non-Indexed Citations (Ovid) to date and EMBASE (Ovid) 1980 to July 2014. Searches used index terms and text words that encompassed the patient group supplemented by terms relating to recurrence or adverse outcome and prognostic factors (see sample MEDLINE search in online supplementary appendix 1).

10.1136/bmjopen-2016-011190.supp1 Supplementary appendix 1

Publicly available trial registers were also searched, such as ClinicalTrials.gov, UK Clinical Research Network Study Portfolio Database (UKCRN), WHO International Clinical Trials Registry Platform and the metaRegister of Controlled Trials (mRCT). Reference lists of all included papers were checked and subject experts were contacted. No restrictions on publication language were applied.

In addition, abstracts from the Conference Proceedings Citation Index (CPCI) were searched in order to capture studies that were not yet fully published.

### Selection/inclusion criteria

#### Study design

Studies of any design (eg, cohorts, randomised controlled trials) or systematic reviews that developed, compared or validated a prognostic model (or clinical prediction rule based on a model) utilising multiple (at least two) predictors to predict the risk of recurrent VTE or adverse outcome (mortality or bleeding) following cessation of therapy for a first unprovoked VTE.

#### Patient group

Patients aged ≥18 years with a first unprovoked VTE where the patient has received at least 3 months treatment with an OAC therapy. Studies with mixed populations (including those outside of remit) were included provided that appropriate data for the defined group of patients was extractable.

#### Setting

Studies in any setting were included.

#### Potential prognostic models

Studies must have reported a prognostic model utilising multiple predictors to predict the risk of recurrent VTE or adverse outcome following cessation of therapy for a first unprovoked VTE. A prognostic model was defined as a combination of at least two predictors within a statistical model, used to predict an individual's risk of outcome (eg, VTE recurrence).

### Study selection

Study selection followed a two-step process. Titles (and abstracts where available) were initially screened by two reviewers independently, using predefined screening criteria. These were broadly based on whether studies, (1) included patients with a first unprovoked VTE, who received a minimum of 3 months OAC therapy and (2) developed or examined prognostic models in relation to individual prediction of VTE recurrence or other adverse outcomes (mortality or bleeding).

Full texts of any potentially relevant articles were then obtained and two reviewers independently applied the full inclusion criteria (see online supplementary appendix 2). Any discrepancies between reviewers were resolved by discussion or by referral to a third reviewer. Portions of non-English language studies were translated where necessary to facilitate study selection and subsequent data extraction. The study selection process was documented using the PRISMA flow diagram. Any relevant systematic reviews identified were screened for further primary studies. Reference management software (Endnote) was used to record reviewer decisions, including reasons for exclusion.

10.1136/bmjopen-2016-011190.supp2 Supplementary appendix 2

### Data extraction

In those articles deemed relevant, data extraction was then conducted independently by two reviewers using an in-depth piloted data extraction form. Disagreements were resolved through discussion or referral to a third reviewer.

Data extraction included the following elements:

- Study characteristics (eg, sample size, country, year);
- Study design characteristics (eg, design, length of follow-up);
- Patient characteristics (eg, summaries of age, sex, family history, treatment details in the sample);
- Candidate predictors considered (eg, any thresholds used for continuous predictors, methods of measurement, timing of measurement postcessation of therapy);
- Outcome measures (eg, recurrence of VTE, mortality, bleeding);
- Statistical methods employed and how predictors included in the analysis were handled (eg, continuous vs dichotomised);
- Prognostic model details, including the final model equation and included predictors; how the model was developed and how it can be used to obtain an individual's risk probability; and any internal and external validation performance statistics for model performance (including discrimination and calibration) together with their CIs.

### Assessment of study quality

The quality (risk of bias) of any studies developing or evaluating a prognostic model was assessed by piloting a version of PROBAST (Prediction study Risk Of Bias Assessment Tool), a tool for assessing risk of bias and applicability of prognostic model studies, that was nearing completion and ready for piloting when this review was undertaken (Wolff R, Whiting P, Mallett S, *et al*, personal communication).

Particular elements were considered in the following domains:

- Patient selection, such as
  What study design was used (eg, prospective),
  If appropriate inclusions and exclusions were used,
  Whether patients had similar disease presentation, or if this was accounted for in analyses.
- Outcomes, such as whether
  The outcome definition was prespecified,
  Included predictors were excluded from the outcome definition,
  The same definition and assessment was used for predictors and outcomes in all patients,
  The outcome was determined blind to predictor information.
- Predictors, such as whether
  The same predictor definitions were used for all patients,
  Predictors were measured blinded to outcome data,
  All predictor information was available at the time the model was intended for use,
  Non-linear associations for continuous predictors were considered and, if undertaken, predictor categorisation was not data driven.
- Sample size, such as
  Whether there was a prespecified sample size consideration for model development accounting for numbers of events and multiple comparisons in selection of predictors,
  Whether all enrolled patients were included in analyses,
  How much data were available for external validation.
- Missing data, including whether
  There was adequate reporting on completeness of data,
  Multiple imputation was considered.
- Statistical analysis, such as
  Handling of continuous predictors,
  Selection of possible predictors irrespective of univariable analyses,
  Whether weights assigned to predictors in the final model's statistical equation related to the same regression coefficients as from the fitted model in the development data.
- Internal and external model validation
  Whether model validations are reported and how these were obtained, in particular whether overfitting and optimism was accounted for using bootstrapping or shrinkage (during internal validation).

### Summarising identified evidence

For each unique model identified, we summarised narratively the evidence available using the data extracted. In particular, we summarised the model development and validation methodology, the included predictors and how they were coded, the specification of the model and how it can be used, whether the model was validated internally and externally (and if so how), and the reported performance of the model in terms of calibration and discrimination. The PROBAST evaluation was used to determine the risk of bias of the model (ie, whether the model is likely to work as intended for the VTE population of interest), with models classed as low, moderate or high risk of bias.

The consistency of development methods used and main findings were examined to identify whether studies at higher risk of bias produced different results and conclusions to those considered to be at low risk of bias.

If multiple studies were found that validated the same prognostic model, it was planned to synthesise calibration (eg, expected/observed events) and discriminatory statistics (eg, C-statistic) using a random-effects meta-analysis,^16^ ^17^ to summarise the model's average performance across different settings and its potential performance in a future setting.

### Relevant articles identified outside of search dates

Two relevant studies were identified since the literature searches were performed; these were published in February 2015^18^ and September 2015, respectively.^19^ The first study is an external validation of the Vienna prediction model using individual participant data (IPD) from five studies, and the later study is an external validation of the updated Vienna prediction model in a prospective multicentre cohort study; both of these will be discussed in detail later as evidence found outside the systematic review searches.^18–20^

## Results

### Quantity of research available

**Figure 1:**
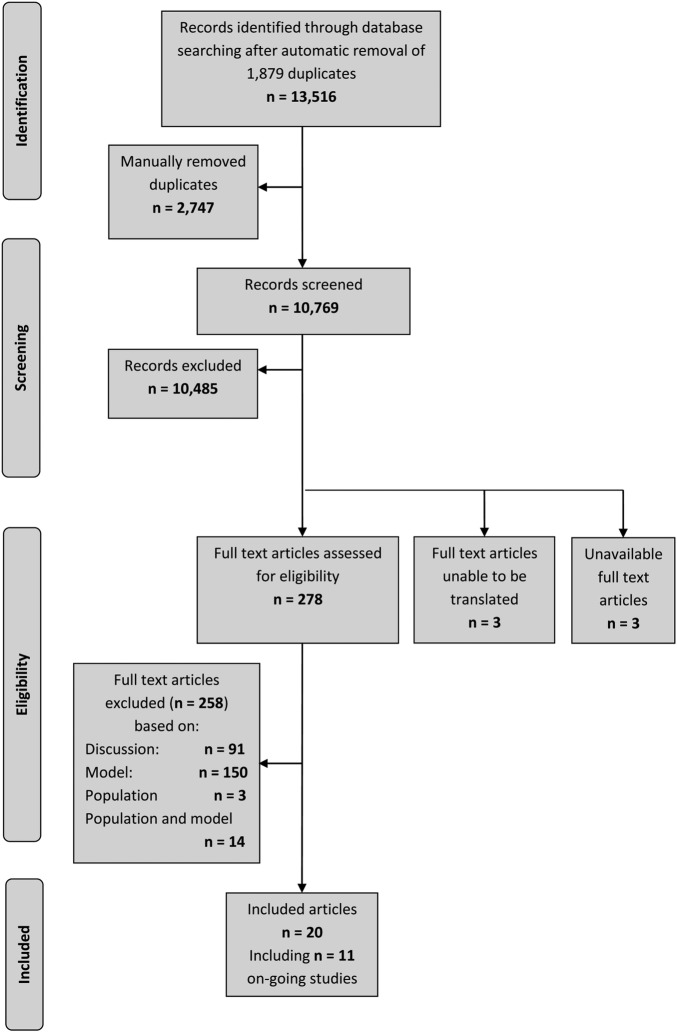
PRISMA flow diagram showing the quantity of research available.

Searching of bibliographic databases resulted in 13 516 records identified after automatic removal of 1879 duplicates. A further 2747 duplicate records were manually removed, leaving 10 769 records to be screened for inclusion. Screening of titles and abstracts identified 10 485 records irrelevant to the review question. Full-text articles were sought for eligibility assessment, three articles were unobtainable from the British library^21–23^ and a further three articles were unable to be translated into English^24–26^ out of 19 non-English language articles (ie, 16 translated). Of the 278 full-text articles assessed for inclusion, 258 articles were excluded with:

- Ninety-one articles excluded as discussion or review articles that did not develop or update a prognostic model;
- One hundred and fifty articles were excluded based on issues related to the model (eg, not for individual prediction, emphasis on the effect of a single predictor, etc);
- Three articles were excluded based on the study population;
- Fourteen were excluded based on population and model issues (see figure 1).

Twenty articles, therefore, met the inclusion criteria after screening, comprising seven ongoing studies,^27–33^ eight conference abstracts,^34–41^ one project record referring to the project this work forms part of^42^ ^43^ and four full-text peer-reviewed articles.^8–10^ ^44^

The authors of the 15 conference abstracts and ongoing studies were contacted to seek additional information. Based on author responses, 13 of the 15 abstracts/ongoing studies were associated with the four full-text articles included. The authors of the remaining two articles (which were both abstracts) did not respond to further enquiry, and so no further publications could be found to supplement the available abstracts.^27^ ^35^ The first abstract related to a study by Raskob *et al*,^35^ based on data from the EINSTEIN extension study,^45^ and aimed to identify a subgroup of patients at high and low risk of recurrent VTE. Further information regarding the study was unavailable from the included abstract; therefore, it was unclear whether a prognostic model was developed and if individual recurrence risk could be predicted from such a model. The second abstract relates to the ongoing VISTA study,^27^ discussed later in the article.

We concentrate now on summarising and critiquing in detail the full-text articles included in the review (and their 13 associated abstracts). First, a brief introduction to the full-text articles and the models developed is given.

### HERDOO2

Rodger *et al*^9^ used conditional logistic regression to develop a prognostic model for use as a clinical decision rule. This suggested that a female patient with less than two predictors (post-thrombotic signs, D-dimer level ≥250 µg/L, body mass index (BMI)≥30 kg/m^2^ or age≥65 years) could potentially safely discontinue OAC therapy after 5–7 months of initial OAC therapy for an unprovoked VTE. A low risk (<3% annual recurrence risk) group of males could not be identified in the study, and therefore Rodger *et al*^9^ recommended that all male patients continue OAC therapy.

### Vienna prediction model

Eichinger *et al*^8^ ^44^ used a Cox proportional hazards model to develop a prognostic model including sex, site of index event and D-dimer as predictors. A nomogram based on the prognostic model was derived to allow easy implementation of the model and can be used to calculate patient's cumulative recurrence rate at 12 and 60 months from cessation of therapy, with estimated 95% CIs.^8^ Another full-text article included in the review describes an update to the proposed Vienna model (discussed later), by recalculating the model at 3, 9 and 15 months after cessation of therapy using new measurements of D-dimer levels at these time points. Eichinger *et al*^44^ used a dynamic prediction approach in the updated model and adapted a Fine-Gray model to allow for the competing risk between recurrence and death (in some of those who restart therapy).

### DASH score

Tosetto *et al*^10^ used a Cox proportional hazards model to develop a prognostic model including predictors for abnormal D-dimer levels (+2 score), age≤50 years (+1 score), male sex (+1 score) and hormone use (−2 score). This proposed score can be used to calculate patient's cumulative recurrence risk at 1, 2 and 5 years from cessation of therapy, with estimated 95% CIs. Tosetto *et al*^10^ suggest that a combined DASH score ≤1 would indicate an annual recurrence risk <5% and therefore indicate that a patient could potentially stop OAC therapy, conversely a DASH score >1 would indicate annual recurrence risk >5% and thus suggest patients should potentially continue OAC therapy.

The population characteristics of the three study populations were broadly similar across predictors measured in all studies (see table 1). The median age of patients in the DASH population was somewhat higher than that of the HERDOO2 and Vienna study populations, and the Vienna study included extended follow-up compared with the other studies, both of which could affect estimates of predictor effects in the models. Throughout this review, these articles will be referred to using the name of the corresponding model developed, as given above (ie, HERDOO2, Vienna and DASH).

**Table 1:** Summary of patient characteristics in included model studies

Model	HERDOO2				Vienna		DASH			
Measurement statistics used	Mean (SD) or frequency (%)				Median (25th, 75th centiles) or frequency (%)		Median or per cent			
Patient characteristic	n	Recurrence	n	No recurrence	n	All	n	Recurrence	n	No recurrence
Age (years)	91	53.6 (14.8)	555	52.3 (17.9)	929	54 (43, 63)	239	63	1579	61
Male proportion	91	63 (69.2)	555	269 (48.5)	929	562 (60)	239	69.40%	1579	48.60%
Site (distal DVT) proportion	91	NA	555	NA	929	164 (17.7)	239	NA	1579	NA
Site (proximal DVT) proportion	91	NA	555	NA	929	327 (35.2)	239	NA	1579	NA
Site (PE) proportion	91	NA	555	NA	929	438 (47.1)	239	NA	1579	NA
BMI (kg/m^2^)	91	30.3 (7.6)	555	28.9 (7.1)	909	27.1 (24.4, 30.1)	*	27.2	*	27.2
D-dimer (µg/L)†	91	383 (738)	555	294 (314)	832	355 (236, 558)	239	67.7%‡	1579	42%‡
Factor V Leiden proportion	91	19 (20.9)	554	81 (14.6)	916	224 (24.4)	239	NA	1579	NA
Duration of OAC (months)	91	5 to 7	555	5 to 7	929	6.6 (6.1, 8.0)	239	6.7	1579	6.8
Duration of follow-up (months)	18 (1, 47)§				43.3 (14.7, 78.5)		22.4			

*BMI data available for 802 participants, no reporting of number of participants by event status.

†D-dimer measured in ng/mL within the DASH article.

‡DASH reported the percentage with abnormal D-dimer, defined as ≥500 ng/mL .

§Follow-up for HERDOO2 presented as mean (range).

NA—the information was not provided for these fields. In particular, both the HERDOO2 and DASH studies did not include patients with distal DVT index events a priori. And the DASH study did not provide figures for the proportion of patients with factor V Leiden, but the percentages of patients with thrombophilia were 23.4% and 20.9% for recurrence and non-recurrence, respectively.

BMI, body mass index; DVT, deep vein thrombosis; OAC, oral anticoagulants; PE, pulmonary embolism.

## Quality assessment and critical appraisal

### Patient selection and outcomes

All of the three articles developed models based on data collected using a prospective design (see table 2), which is ideal for prognostic modelling as predictor information can be collected blind to patient outcome. Across all three articles, recurrent VTE (at various predicted time points) was the primary outcome (see table 2), and was objectively confirmed and independently adjudicated. Detection bias was limited in all three articles by prespecification of outcome definitions, with the same definition and assessment used for all patients (within each study), meaning systematic differences in the determination of outcomes were avoided.

**Table 2:** Study characteristics

Model	HERDOO2	Vienna	DASH
Year of publication	2008	2010	2012
Country	Four countries (unspecified)	Austria	Austria, Canada, Italy, Switzerland, UK, USA
Study setting	12 tertiary care centres, patients enrolled between October 2001 and March 2006	Recruited from 4 thrombosis centres in Vienna between July 1992 and August 2008	Patient-level meta-analysis of previously published studies (11)
Study design	Prospective cohort study	Prospective cohort study	Individual patient data from 7 prospective studies
Clinical outcome	Recurrent VTE	Recurrent VTE	Recurrent VTE
Key prediction time points (months)	12 months	12, 60 months	12, 24, 60 months
Total sample size	646	929	1818
Events	91	176	239

VTE, venous thromboembolism.

The inclusion/exclusion criteria used in the three articles is summarised in table 3, and common criteria included the exclusion of patients with high-risk thrombophiliac conditions, patients <18 years old and patients treated with <3 months OAC therapy.

**Table 3:** Study inclusion/exclusion criteria

Model	HERDOO2	Vienna	DASH
Inclusion criteria	First unprovoked VTE Received OAC 5–7 months No recurrent VTE on treatment	First unprovoked VTE Age≥18 Received OAC≥3 months	First unprovoked VTE Including thrombophilic blood abnormalities where there were no other VTE risks
Exclusion criteria	Age<18 Deficiency in antithrombin, protein C or S Presence of lupus anticoagulant Already discontinued OAC Geographically inaccessible to follow-up Not proximal DVT or PE index event	Deficiency in antithrombin, protein C or S Presence of lupus anticoagulant Presence of cancer	Known antiphospholipid antibodies Antithrombin deficiency Not proximal DVT or PE index event

DVT, deep vein thrombosis; OAC, oral anticoagulants; PE, pulmonary embolism; VTE, venous thromboembolism.

All articles only included patients with a first unprovoked VTE, but definitions of unprovoked varied somewhat (see table 4). The HERDOO2 and DASH models both included patients with hormone intake at time of index event, while the HERDOO2 model also included pregnancy-associated VTE at index event within its definition of unprovoked VTE. The DASH model study justifies including hormone intake as unprovoked because some evidence suggests hormone therapy is a weak predictor for VTE recurrence.^10^ ^46^ However, evidence suggests that these risk factors are acquired,^3^ ^4^ and inclusion of patients outside the unprovoked population might therefore lead to biased conclusions about predictor effects.

**Table 4:** Unprovoked venous thromboembolism definition across studies

Model	HERDOO2	Vienna	DASH
Not provoked by			
Trauma	X	X	X
Surgery	X	X	X
Cancer	X	X	X
Pregnancy	–	X	X
Immobility	X	–	X
Hormone intake	–	X	–

### Predictors

The three studies investigated a wide variety of candidate predictors, including clinical and laboratory predictors. There was some overlap between models (see table 5), with D-dimer, age and sex being the most commonly included predictors. The Vienna model avoided the categorisation of continuous candidate predictors, while the DASH model investigated patient age in prespecified quartiles, to allow for non-linear associations between age and recurrence risk. The HERDOO2 model in contrast performed χ^2^ testing to identify the optimal threshold to dichotomise every continuous predictor under consideration.

**Table 5:** Predictors included in final model

Model	HERDOO2	Vienna	DASH
Predictors included			
D-dimer	X	X	X
Age	X	–	X
Sex	–	X	X
BMI	X	–	–
Post-thrombotic signs	X	–	–
Site of index event	–	X	–
Hormone therapy	–	–	X

BMI, body mass index.

Data-driven analyses are known to incite reporting biases, where optimal thresholds are reported without any clinical meaning.^47^ Dichotomisation of continuous predictors is also methodologically poor, as it seeks to separate patients risk into two categories, treating all those above the threshold as having the same constant risk (and similarly for those below the threshold), which is unrealistic in practice.^47^

All models also allowed for site of index event in some way. Both the HERDOO2 and DASH models excluded patients with distal DVT index events from their studies,^9^ ^10^ which is important risk stratification in itself (ie, both models are not applicable to patients with distal DVT events). Only the Vienna model included such patients and adjusted for site of index event as a predictor in the model (see table 5). The Vienna models predicted risks reflect the low risk of recurrence associated with distal DVT index events, and provides an estimate of risk (where the other models do not) which may be a helpful tool in consultation with patients and confirm treatment decisions.^14^

### Sample size

**Figure 2:**
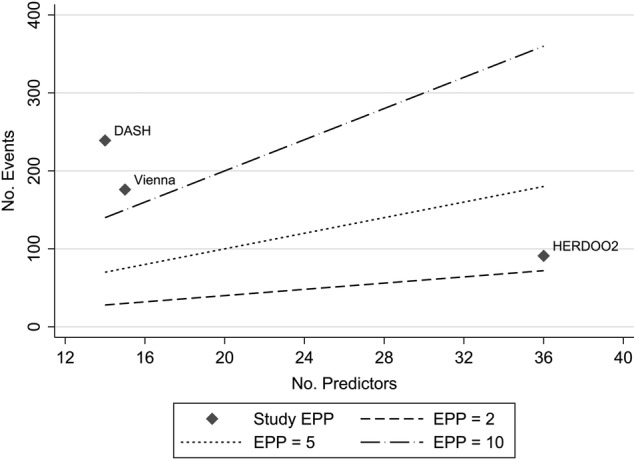
Events per predictor (EPP) for included studies, based on total sample size and number of predictors. NB: lines represent number of events required to maintain EPP=x for given number of predictors.

The HERDOO2 model was markedly underpowered, having collected information on 69 predictors and considered at least 36 of these. There were only 91 recurrent events (see table 2), meaning the HERDOO2 model only had around 2.5 events per predictor (EPP), assuming complete predictor availability for all patients. Evidence shows that an EPP<10 can lead to bias in estimates of predictor effects and their SEs, as well as the coverage of CIs, with EPP=2 showing severe biases.^48^ ^49^ This may then cause overfitted models (ie, models that include inappropriate predictors or predictor effects that are too large). The Vienna and DASH models investigated 15 and 14 candidate predictors, respectively, with 176 and 239 total events, respectively (see table 2). Following the same rule of thumb (EPP<10)^48^ ^49^ and assuming complete predictor availability for all patients (ie, no missing data), the Vienna (just) and DASH models therefore had sufficient numbers of events to assess the predictors of interest with appropriate statistical power (see figure 2).

### Missing data

**Figure 3:**
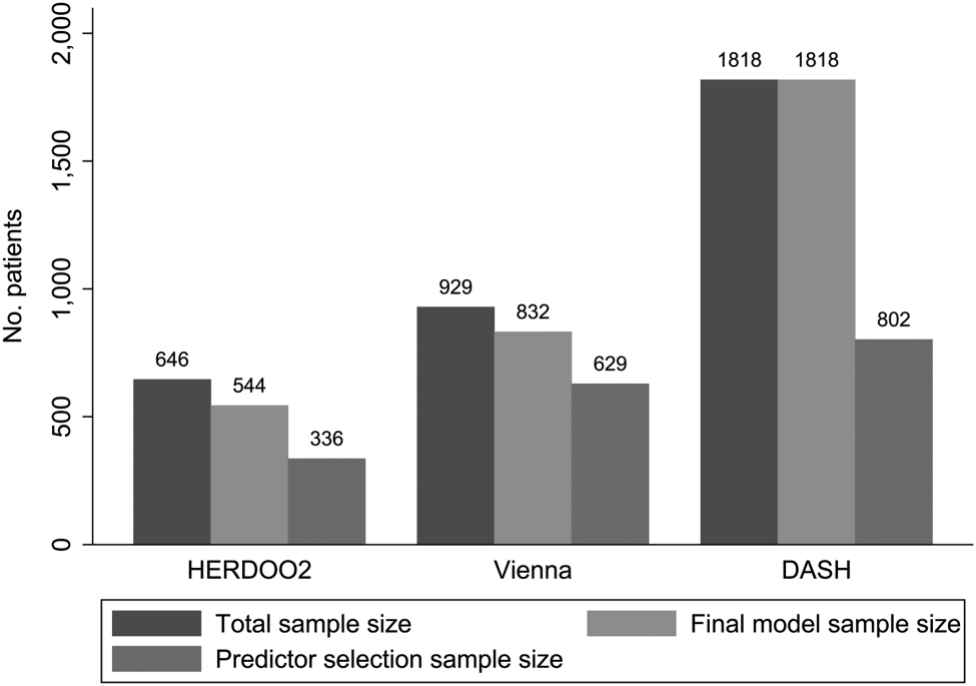
Final model sample size compared to total & selection sample size. Final model sample size=total sample minus patients with missing information in any predictor included in the final model; Predictor selection sample size=total sample size minus patients with missing predictor information in any predictor considered for inclusion in the model using a selection procedure.

All of the three included studies suffered from some degree of missing predictor information, and used a complete case analysis to overcome this issue. The presence of missing predictor data will further reduce the apparent EPP discussed above (see figure 2). Each study also used a selection procedure meaning more predictors were considered, resulting in a higher proportion of missing predictor data. For example, the Vienna prediction model considered peak thrombin as a predictor, for which 300 out of 929 patients had missing predictor information.^8^ Similarly, the DASH model considered predictors including BMI, for which only 802 out of 1818 patients had complete predictor information.^10^ Meaning that the predictor selection process included a massively reduced sample size compared with the final model complete case data used, which may have increased the chance of spurious predictor–outcome relationships (see figure 3).

No methods to assess the impact of this missing predictor information were used, and in the Vienna and DASH models, the number of missing recurrent events was not reported, so no assessment of the impact on statistical power (nor EPP) could be made accurately. A complete case analysis in the presence of missing data does not represent the entire population, and reduces sample size making predictor effects only pertinent to a specific subgroup of the population with no missing data. Multiple imputation (MI) can be used as a sensitivity analysis to assess the impact of missing data on the performance of the model. MI using chained equations imputes missing predictor information from a posterior distribution based on the observed data^50^; it increases sample size, power and may improve the generalisability of the model.

### Statistical analysis

A Cox proportional hazards model was used to develop both the Vienna and DASH models, which appropriately accounts for censoring of patients in the analysis of time-to-event outcomes such as recurrence. The HERDOO2 model used a conditional logistic model for analyses, which does not account for the censoring of patients over time and the variable lengths of individual follow-up.

All studies recruited patients from different centres or countries (see table 2); however, only one (DASH model) stratified by source in their analyses. Stratification accounts for heterogeneity in the baseline recurrence risk in different patient groups. Ignoring the clustering of patients within centres or countries could lead to poor model calibration (where model predictions do not closely fit observed recurrence rates) and/or biased predictor effects,^51^ ^52^ and thus could diminish performance in a new setting. The DASH model did not propose how to implement the model in practice; where models are stratified, there are several options for implementation^12^ ^52^ (eg, use a single intercept related to one of the centres).

The HERDOO2 model excluded predictors from multivariable analysis where univariable analysis yielded p≥0.2; this predictor selection strategy was therefore completely data driven, which could lead to potential bias in results, with predictor effects that may be important in combination being excluded. The DASH model also excluded some predictors from multivariable analysis on the basis of univariable results. Univariable analyses are not recommended for decisions about inclusion of predictors in a multivariable model.^53^

Both the Vienna and DASH models used bootstrapping and shrinkage methods to adjust predictor coefficients for overoptimism, but the HERDOO2 development did not account for optimism in predictor estimates. The use of optimism correction methods reduces overfitting by reducing the magnitude of predictor effects, to help ensure the model performance is more accurate in a new patient population.

The specification and application of the proposed models was described in various ways across the studies. Both the Vienna and DASH models were presented well, with an indication of how the predictors are combined to calculate a patient's recurrence risk at a specific time point. Both provided cumulative recurrence risk at specific time points after cessation of therapy including an estimate of the uncertainty surrounding these estimates (95% CIs). This information could be used to direct the decision-making process, informing clinicians and patients of the individual's level of risk, and therefore allowing individualised treatment strategies. However, neither reported any estimate or parameterisation of the baseline hazard function, which would be required for full external validation of the model in a new setting.^54–56^

Conversely, the HERDOO2 model derived a clinical decision rule splitting patients into those with less than two predictors (from their model), and those with greater than two predictors, suggesting that one group should continue OAC therapy, while the other could safely stop. The HERDOO2 model did not report individuals risk at specific time points, only that fewer than two predictors would indicate a <3% annual risk of recurrence. This therefore does not allow clinicians or patients to make decisions based on their preference of recurrence risk threshold, limiting the applicability of the decision rule if a value other than 3% was of interest.

### Model validation

Model performance was evaluated using internal validation in all the studies, but none reported an external validation.^8–10^ Internal validation was reported in terms of calibration and discrimination with the Vienna and DASH models presenting both (though not for the simplified Vienna nomogram, which constitutes the final model). The HERDOO2 model presented neither calibration nor discrimination statistics. The performance statistics reported are given in table 6. Apparent C-statistics (which represent the discriminatory performance within the development data without adjustment for optimism using, eg, bootstrapping) are between 0.65 and 0.72 for the different models, indicating moderate discrimination ability; however, apparent performance is likely to be optimistic. The Vienna model also presented a bootstrap adjusted C-statistic (accounting for optimism), of 0.646 for predictions at 5 years postcessation of therapy, indicating a small reduction after accounting for optimism. The Vienna and DASH models also provided a bootstrap optimism-adjusted calibration slope (or uniform shrinkage factor), which showed moderate calibration performance of 0.88 for the Vienna model, and strong performance of 0.97 for the DASH model (with 1 indicating perfect calibration). Both the Vienna and DASH models used their shrinkage factor to adjust the predictor effect values in their final model, to adjust for the overoptimism.

**Table 6:** Internal validation performance statistics

Model	Calibration slope*	Apparent discrimination†	Bootstrap-adjusted discrimination‡
HERDOO2			
Model for use (score)	–	–	–
Development model (β terms)	–	–	–
Vienna			
Model for use (nomogram)	–	–	–
Development model (β terms)	0.88	0.651	60 months=0.646
DASH			
Model for use (score)	–	0.71	–
Development model (β terms)	0.974	0.72	–

*Bootstrap calibration slope.

†C-statistic based on development data.

‡C-statistic based on bootstrap internal validation.

External validation is the true indication of model performance in the wider population, as a model validated within its development data set will give optimistic performance statistics.^11^ ^54^ ^57^ External validation studies are currently being undertaken to validate both the HERDOO2 model^28–30^ and the Vienna model,^27^ which will provide a more robust indication as to the overall performance (in terms of calibration and discrimination) of these models in new patient populations where they are intended for use. The REVERSE II study is a randomised trial aiming to compare the use of the HERDOO2 model to decide on cessation of OAC therapy, compared with standard practice.^28^ ^30^ The VISTA study is a randomised trial comparing the use of the Vienna model to decide on treatment duration, with usual care where treatment duration is based on physicians judgement.^27^

### Update to the Vienna prediction model

The authors of the Vienna model also recently developed an update to the original Vienna model, with the aim of predicting recurrence risk at later time points using updated D-dimer measurements.^44^ New D-dimer measurements were taken at 3, 9 and 15 months postcessation of therapy, with analyses showing a slight decrease in HRs for the effect of log D-dimer over time (though the 95% CIs remained similar).^44^ Three new nomograms were developed for use in practice to predict recurrence risk at 12 and 60 months from time of new D-dimer measurement. A web-based calculator was also developed by the authors allowing prediction of recurrence risk at any integer month after baseline (3 weeks) and up to 15 months postcessation of therapy.

The updated model was adjusted for optimism using leave-one-out resampling to calculate shrinkage factors for 3, 9 and 15 months of 0.79, 0.81 and 0.7, indicating moderate calibration of the model but reduced performance compared with the original Vienna model (optimism-adjusted calibration slope=0.88). Discriminatory performance for 5-year predictions at each new time point showed a small reduction in performance compared with the original model (optimism-adjusted area under the curve (AUC) values were 0.61, 0.61 and 0.58, for 3, 9 and 15 months, compared with AUC=0.646 for the original model).^8^ ^44^ The updated Vienna model expands the earlier model by allowing dynamic prediction of recurrence risk over time, but while the earlier Vienna model has recently been externally validated,^18^ this model has not been externally validated to date, and shows inferior internal validation performance statistics compared with the original model.

### Relevant articles identified outside of review search dates

Subsequent to the completion of our review searches, two additional highly relevant studies were identified.^18^ ^19^ The first was an external validation of the Vienna prediction model using IPD from five studies, which aimed to assess the performance of the Vienna model in terms of discrimination and calibration in a new population.^18^ ^20^

The study reported that the derivation and validation populations were homogeneous after removal of patients with provoked VTE and those with missing predictor information.^18^ Discrimination was calculated using the C-statistic for comparison to the original Vienna model, with a C-statistic in the validation cohort of 0.626 compared with 0.646 (the optimism-adjusted discrimination—see table 6) for the derivation data, indicating a reduction in the discriminatory performance of the model in a new setting.

The true calibration of the model in the validation data could not be assessed without the baseline hazard function.^54–56^ ^58^ As the original Vienna model was developed using a Cox model which does not parameterise the baseline hazard function, this meant that assumptions about the shape of the baseline hazard function had to be made.^18^ ^54–56^ The authors recalibrated the Vienna model assuming a Weibull distribution; however, because this new component of the model was developed, this new model would itself require further external validation.^54–56^ As the authors could not use the Cox model directly to predict survival probabilities (due to the lack of baseline hazard function), they could only assess weak calibration using the prognostic index to make predictions within the validation data.^54^ ^58^ Comparison of observed and expected survival probabilities in five risk groups showed a general trend for the Vienna model to underpredict the risk of VTE recurrence at 12 months postcessation of therapy.^18^ It should be noted that the study did not validate the simplified Vienna nomogram proposed for use in practice.^18^ ^54^

The second study identified was an external validation of the updated Vienna model in a prospective multicentre cohort study.^19^ The study aimed to validate the updated model in elderly patients over 65 years old, and assessed the model’s performance in terms of discrimination and the proportion of recurrent events between high-risk and low-risk patients defined by the model. The study found no difference between the proportion of recurrences in the low-risk versus high-risk groups (where recurrence risk <6.2% 12 months was defined as low risk). Discriminative performance was poor at both 12 and 24 months, with C-statistics of 0.39 (95% CI 0.25 to 0.52) and 0.43 (95% CI 0.31 to 0.54), respectively.

The study suffered from a very low number of events, 17 and 26 by 12 and 24 months, respectively. Therefore, the conclusions of the study should be interpreted with caution, as it is known that small validation samples tend to show poor calibration and discrimination performance, with current recommendations indicating that validation sample sizes should be a minimum of 100 events and preferably ≥200 events.^58^ ^59^ Also there were several distinct differences between the derivation patient population for the updated Vienna model and the validation sample used by Tritschler and colleagues which naturally led to heterogeneity in model performance. In particular, the validation study used a much older population (median (IQR) age 74 (69–79.8) vs 54 (43–63) in the derivation population). This also led to differences in D-dimer levels, with the elderly patients in the validation study having much higher D-dimer levels (median (IQR) D-dimer 1022 (607–1755) vs 355 (236–558) in the derivation population). Further to this, women in the validation study appeared to have much greater risk of recurrence than men, which is discordant with current evidence suggesting that men are between 1.5 and 2 times more likely to suffer a recurrence.^8–10^ ^60^ ^61^ These differences in baseline characteristics may mean that the predictor effects in the updated Vienna model are miscalibrated when applied in this new population, leading to the poor performance seen in the validation study.

### Quality assessment and risk of bias summary of HERDOO2, Vienna and DASH models

Quality assessment based on an early version of the PROBAST tool showed that there was evidence throughout the included studies of a moderate-to-high risk of bias (see table 7), predominately because of a lack of external validation (see tables 6 and 7). The HERDOO2 model development suffered high risk of bias, and some marked methodological issues, including the choice of analysis model, substantially underpowered analyses, data-driven categorisation of predictors, lack of adjustment for optimism and poor presentation of the model for use (see table 7). In contrast, the Vienna prediction model and DASH score were considered generally methodologically sound in terms of their development. Both had statistical power to investigate their candidate predictors, accounted for optimism in their selection procedures, and the Vienna study assessed continuous predictors without categorisation and loss of information (though the DASH study did categorise continuous predictors). Both studies presented their proposed models more clearly than the HERDOO2 model, indicating the recurrence rate associated with predictor values and the uncertainty around those estimates. However, predictions were only provided for particular, discretised values of risk, for example, both models provide predictions for only a small selection of time points (Vienna model for 12 and 60 months post-therapy, DASH score for 1, 2 and 5 years from cessation of therapy); both models only provide 95% CIs for a small selection of predicted annual recurrence rates.

**Table 7:** Quality considerations for included studies

Model	HERDOO2	Vienna	DASH
Use of a selection procedure?	Yes	Yes	Yes
Adjustment for optimism in selection procedure?	No	Yes	Yes
Events per predictor >10?	No	Yes	Yes
Appropriate type of model?	No	Yes	Yes
Modelled continuous predictors as linear/non-linear?	No	Yes	No
Considered multiple imputation to handle missing data?	No	No	No
Adjustment for optimism in internal validation?	Yes	Yes	Yes
Reported discrimination?	No	Yes*	Yes
Reported calibration?	No	Yes*	Yes*
Were final model predictor weightings related to regression coefficients?	Yes	Yes	Yes
Internal validation?	No	Yes*	Yes
External validation?	No	Yes*	No
Risk of bias?	High	Moderate	Moderate
Key reason for decision	No external validation/several quality issues	External validation	No external validation

*Not for the nomogram/score used in practice.

Despite being of generally good methodological quality for development, both Vienna and DASH were classed at moderate risk of bias due to a lack of sufficient external validation (see table 7). The DASH score has received no external validation, and any such future validation should account for the method of implementation, which was not proposed by the authors. The Vienna model has now been externally validated (as discussed above), but issues remain because: (1) validation performance was shown to be lower than expected and uncertainty was high;^18^ (2) a new Weibull model component was added, which itself requires additional validation;^54–56^ (3) the nomogram version of the model, which is the most used, was not validated^54^ and (4) validation of the updated dynamic Vienna prediction model in a new population also indicated poor performance. Thus, until further external validation is undertaken and the results of ongoing validation trials are available, the true performance in new populations cannot be ascertained.

## Discussion

This systematic review of prognostic models for VTE recurrence risk identified four full-text articles developing three independent prognostic models.^8–10^ ^44^ A critique of the included studies described and identified the strengths and weaknesses of the studies with a particular focus on methods of patient selection, outcome reporting, predictor selection, sample size, model development and validation.

First, a key finding was the different definitions of unprovoked VTE across the included studies (see table 4). The Vienna model study excluded patients with index events provoked by use of female hormones, such as the contraceptive pill or hormone replacement therapy, while the HERDOO2 and DASH studies defined index events related to hormone use as unprovoked. Risk factors consistently defined as provoking across the studies included surgery, trauma, immobility and pregnancy (see table 4). The use of varying definitions to describe the unprovoked population creates confusion as to which population the proposed models are applicable to. Further research in developing prognostic models to predict recurrence risk in an unprovoked population should aim to use a standard, consistent definition for the population, excluding patients with acquired/removable risk factors,^4^ to ensure that model predictions are reliable for intended patients. Given the definition of unprovoked VTE used in the DASH and HERDOO2 studies, the proposed models may not be applicable within an unprovoked population.^3^ ^4^

Across the included studies, various predictors were included within the proposed final models, with sex, site of index event and D-dimer level (post-therapy) being included consistently within all three models, indicating strong evidence of an association with recurrence risk (see table 5). As such any future model development should consider including these predictors, as they appear prognostic for recurrence risk, and thus evaluate new predictors in addition to these. Indeed the discrimination performance shown in current models was moderate at best, and therefore any new model would ideally include additional predictors to improve the discriminatory performance statistically, though a parsimonious model may better facilitate implementation in practice.^14^ ^62^ While it has been discussed that the effect of D-dimer as a predictor may be dependent on the method/assay used, previous research has investigated the link between variability in D-dimer assays and recurrent VTE, and found that varying assays do not alter the prognostic value of D-dimer in predicting recurrence.^20^

After evaluation of the models' development and validation criteria, all models were labelled with at least a moderate risk of bias. This was mainly due to a lack of robust external validation, which is essential as prognostic model performance is known to be optimistic when evaluated on the same data used to develop the model.^11^ The HERDOO2 model development was classed at high risk of bias, as—alongside no external validation—it had methodological concerns, including the choice of analysis model, substantially underpowered analyses, data-driven categorisation of predictors, lack of adjustment for optimism and the presentation of the model for use.^9^ The Vienna model and DASH score were methodologically sound, as they had adequate statistical power to investigate their candidate predictors, accounted for optimism in their selection procedures, the Vienna model assessed continuous predictors without categorisation and loss of information, and both presented their proposed models clearly.^8^ ^10^ However, until further external validation is performed, the true performance in new populations cannot be ascertained.

The new external validation study for the Vienna model adds important information on the applicability of the model in practice. The study shows that the ability of the model to identify those at high and low risk of recurrence is weaker in a new population outside of the derivation data set.^18^ However, it is important for the Vienna model to undergo further validation, because the validation study related to the fitted model (ie, the prognostic index from the fitted Cox model), and not the nomogram (which potentially used a simplified set of regression coefficients) which was recommended for use. The updated dynamic Vienna prediction model has now also been externally validated in an elderly population, which showed poor discriminatory performance, but suffered from small validation sample sizes and large variations in case-mix from the original model’s intended population.^19^

To our knowledge, this is the first systematic review identifying prognostic models for VTE recurrence risk in the unprovoked population, and as such it is a strength of the study that a robust systematic methodology was used, which yielded a large amount of potential research, making it unlikely that any relevant study was not included. However, a limitation of the review is that at this time, the searches performed are somewhat out of date, though efforts have been made to include relevant articles identified since the search end dates. A limitation of this review is that the conclusions and quality classifications for the prognostic models discussed in this article are based on the reporting standards of the original articles. Further, we were unable to perform a quantitative analysis of the identified articles due to a lack of homogeneity in many areas, including the predictors used, model types and study populations.

In conclusion, currently available models to predict risk of recurrent VTE in an unprovoked population have several limitations. In particular, sufficient external validation has not yet been performed for two of the available models and we recommend further validation studies are required before the models are implemented into practice. Even then the impact of the model on clinical decision-making and, crucially, patient outcomes should be evaluated through a randomised trial, ideally, or health economic modelling exercise.^11^ ^43^ Any new models should try to build on the existing work, ensure external validation in multiple populations, transparency in reporting of model development as outlined in the TRIPOD statement,^13^ and finally improved statistical analyses to ensure model predictions are more robust.
