# Supplementary material for: Systematic review of prognostic models for recurrent venous thromboembolism (VTE) post-treatment of first unprovoked VTE
Source: BMJ Open. 2016 May 6;6(5):e011190. doi: 10.1136/bmjopen-2016-011190 (PMC4861103; doi:10.1136/bmjopen-2016-011190)
Supplement: Supplementary appendix 1 [file bmjopen-2016-011190supp_appendix1.pdf]

Appendix 1: Example search strategy for MEDLINE

Database: MEDLINE (Ovid) 1946 to July Week 3, 2014
Search strategy:
1. exp Venous Thromboembolism/
2. Pulmonary Embolism/
3. exp Venous Thrombosis/
4. (vte or dvt or pe).ti,ab.
5. deep vein thrombosis.ti,ab.
6. pulmonary embolism.ti,ab.
7. venous thrombo$.ti,ab.
8. or/1-7
9. (recurrence or recurr$ or re-occur$).ti,ab.
10. Recurrence/
11. exp Death/
12. (death$ or mortality).ti,ab.
13. Mortality/
14. clot$.ti,ab.
15. Hypertension, Pulmonary/
16. pulmonary hypertension.ti,ab.
17. post thrombotic syndrome.ti,ab.
18. PTS.ti,ab.
19. or/9-18
20. “Predictive Value of Tests”/
21. predict$.ti,ab.
22. exp Risk/
23. risk$.ti,ab.
24. prognos$.ti,ab.
25. or/20-24
26. exp Anticoagulants/
27. (anti-coagul$ or anticoagul$ or warfarin or acenocoumarol or coumadin or coumarin or phenprocoumon or sintrom or sinthrome or jantoven or marevan or waran or nicoumalone or dicoumarol or dicumarol).ti,ab.
28. (phenindione or dabigatran or ximelagatran or apixaban or rivaroxaban or edoxaban or azd0837 or ly517717 or ym150 or betrixaban or idraparinux).ti, ab.
29. or/26-28
30. 8 and 19 and 25 and 29
