# Supplementary material for: Systematic review of prognostic models for recurrent venous thromboembolism (VTE) post-treatment of first unprovoked VTE
Source: BMJ Open. 2016 May 6;6(5):e011190. doi: 10.1136/bmjopen-2016-011190 (PMC4861103; doi:10.1136/bmjopen-2016-011190)
Supplement: Supplementary appendix 2 [file bmjopen-2016-011190supp_appendix2.pdf]

Appendix 2: Full text inclusion criteria

CRITERIA	Yes	C/T	No	Prognostic model
**Reviews & Discussions**				Does the study do more than just discuss a model
**Population**				Are patients at least 18 years old
				Could the population or a defined subpopulation be considered as unprovoked (if not why not)
				Can we identify results specifically for the unprovoked population
				Did patients receive at least 3 months treatment with either a vitamin K antagonist or an oral anticoagulant
**Outcome**				Does the model predict least one of: recurrence/mortality/bleeding/QoL
**Models**				Does the model aim to do more than assess a single factor adjusted for other things
				Is the model used to predict risk of one of the above outcomes
**DECISION**				
**Exclude** *with reason*				
Does the study include an economic evaluation of a model?				
**Comments**				
**IF INCLUDED:**	**Yes**	**C/T**	**No**	**Factors**
**If the study is included, what is their definition of unprovoked?**				Major surgery
				Lower limb trauma
				Use of oral contraceptive pill or hormone replacement therapy
				Pregnancy
				Significant immobility
				Cancer
				Thrombophillia (e.g. antiphosphlipid syndrome, factor V leiden etc.)

Unprovoked = no history (within 3 months) of major surgery; lower limb trauma e.g. fracture, cast; use of the combined oral contraceptive pill or hormone replacement therapy ; pregnancy; significant immobility e.g. confined to bed for 3 days; cancer

A "yes" in all categories under CRITERIA indicates to include a study, any "no's" indicate exclusion
